# A meta-analysis of serum uric acid and diabetic nephropathy risk in type 2 diabetes

**DOI:** 10.3389/fendo.2025.1651446

**Published:** 2026-01-26

**Authors:** Jing Zhao, Lingzhu Zhao

**Affiliations:** 1 Department of Nephrology & Hemodialysis Center, Xi’an Ninth Hospital, Xi’an, Shaanxi, China; 2 Department of Neurology-Unit 1, Xi’an Ninth Hospital, Xi’an, Shaanxi, China

**Keywords:** type 2 diabetes mellitus, serum uric acid, diabetic kidney disease, intervention, meta-analysis

## Abstract

**Objective:**

To explore the correlation between serum uric acid (SUA) levels and the risk of diabetic kidney disease (DKD) in patients with type 2 diabetes mellitus (T2DM), and to evaluate the potential clinical implications of uric acid-lowering interventions.

**Methods:**

Relevant studies on the relationship between serum uric acid levels and the risk of DKD in patients with type 2 diabetes mellitus were collected by systematically searching databases such as PubMed, Web of Science, and Cochrane Library. The quality of the included studies was evaluated using the Cochrane risk-of-bias assessment tool, and Meta-analysis was performed using RevMan 5.3 software. The primary outcome indicators included the incidence of DKD, the odds ratio (OR) and 95% confidence interval (CI) of the relationship between serum uric acid levels and the risk of DKD.

**Results:**

After retrieval and screening, 8 randomized controlled trials (RCTs) (with a sample size of 491) were included in the Meta-analysis. The results showed that the estimated glomerular filtration rate (eGFR) in the hyperuricemia group was lower than that in the normal group (MD = 4.40, 95% CI [0.66, 8.14], *P* = 0.02), with low heterogeneity; the risk of DKD was significantly increased (OR = 1.85, 95% CI [1.52, 2.26], *P*<0.001), with moderate heterogeneity (I²=49%). Sensitivity analyses confirmed the robustness of these findings, though the limited sample size and moderate heterogeneity suggest caution in generalizing the results.

**Conclusion:**

Elevated serum uric acid levels are significantly associated with an increased risk of DKD in patients with type 2 diabetes mellitus. Monitoring serum uric acid levels may help to identify high-risk individuals for DKD at an early stage and provide a reference for clinical intervention.

## Introduction

Type 2 diabetes mellitus (T2DM) is one of the most common chronic metabolic diseases globally, and its prevalence has been continuously increasing with the aging population, rising obesity rate, and changes in lifestyle (1). T2DM not only seriously affects the quality of life of patients but may also lead to a variety of severe complications, among which microvascular complications are particularly common (2). Diabetic Kidney Disease (DKD) is one of the most severe microvascular complications of T2DM and is also the main cause of End-Stage Renal Disease (ESRD) (3). Statistically, approximately 20%-40% of T2DM patients will eventually develop DKD. Once patients enter the ESRD stage, they need to rely on dialysis or kidney transplantation to maintain life, which significantly increases the medical burden and socio-economic costs (4). Therefore, early identification of high-risk factors for DKD and implementation of intervention measures are crucial for delaying the progression of the disease.

Although SUA’s association with DKD is well-documented, two critical gaps remain: Most evidence comes from observational studies, leaving causal inferences uncertain; Few randomized controlled trials (RCTs) have examined whether uric acid-lowering interventions can mitigate DKD risk. This meta-analysis focuses exclusively on RCTs to strengthen causal evidence and highlight the need for intervention trials. Some studies have shown that elevated serum uric acid (SUA) was an independent risk factor for DKD and is significantly associated with the decline of renal function and the progression of proteinuria. Nevertheless, other studies believe that elevated SUA may be a secondary manifestation of renal function impairment rather than a direct pathogenic factor (5–7). In addition, differences in population characteristics, uric acid measurement methods, and DKD definition criteria among different studies have led to inconsistent conclusions. Therefore, there is an urgent need to integrate existing evidence through systematic reviews and Meta-analyses to more comprehensively evaluate the association between SUA and the risk of DKD. However, the clinical utility of SUA as a biomarker and the therapeutic potential of uric acid-lowering strategies remained understudied. This meta-analysis aims to consolidate existing evidence from RCTs and highlight gaps for future research, particularly in evaluating interventions targeting SUA to improve renal outcomes.

This study aims to systematically evaluate the correlation between SUA levels and DKD risk in T2DM patients through meta-analysis of RCTs, providing evidence for clinical intervention.

## Materials and methods

### Inclusion and exclusion criteria

Inclusion Criteria: 1) Study type: Only RCTs were included. 2) Study subjects: Patients with type 2 diabetes aged ≥18 years were included. 3) Intervention measures: The studies were required to report serum uric acid levels. 4) Outcome indicators: The incidence or risk of diabetic nephropathy was required to be reported.

Exclusion criteria: 1) Non-randomized studies: Observational studies, case-control studies, and retrospective studies were excluded. 2) Comorbidities: Patients with comorbid other kidney diseases or severe cardiovascular diseases were excluded. 3) Incomplete data: Studies without providing key data were excluded.

### Search strategy

Database selection: PubMed, Web of Science, EMBASE, Cochrane Library and Medline. The retrieval time range was from the establishment of each database to October 2024. The main keywords include “Type 2 Diabetes Mellitus”, “Serum uric acid level”, “Diabetic Kidney Disease”, and “Risk”. Boolean logical operators (AND, OR) were used to optimize the retrieval results. For example: ((((((Type 2 Diabetes Mellitus) OR (T2DM)) AND (Serum Uric Acid)) OR (Hyperuricemia)) AND (Diabetic Kidney Disease)) OR (DKD)) OR (Diabetic Nephropathy).

### Literature screening and quality evaluation

The retrieved literatures were screened by two independent researchers. Studies that did not meet the criteria were excluded, and full-text reading was carried out for the eligible studies. The quality of the included studies was evaluated using the Cochrane risk-of-bias assessment tool.

### Statistical analysis

Meta-analysis was performed using RevMan 5.3 software. Count data were expressed as odds ratio (OR), and measurement data were expressed as mean difference (MD), with 95% confidence interval (CI) marked. When *P*>0.1 and I²<50%, a fixed-effects model was adopted; when *P*<0.1 and I²>50%, a random-effects model was used. Meanwhile, a funnel plot was drawn to evaluate publication bias. To quantitatively assess publication bias, Egger’s linear regression test was performed for each outcome using Stata 17.0, with p<0.10 indicating significant asymmetry.

## Results

### Literature retrieval results

**Figure 1:**
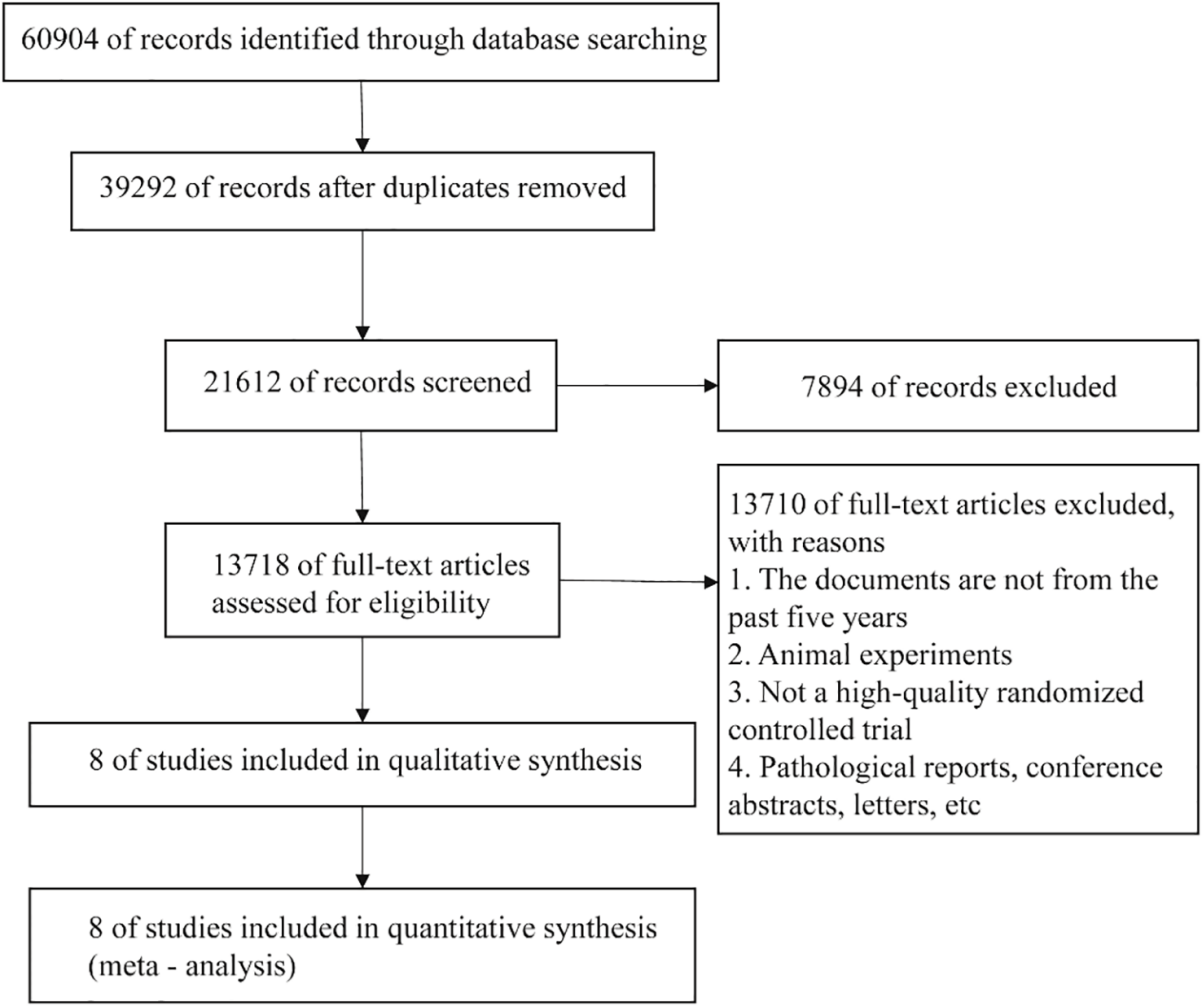
Flowchart of literature retrieval.

A total of 60,904 literatures were obtained through retrieval in relevant databases. According to the inclusion and exclusion criteria, 8 literatures were finally included (**Figure 1**).

### Basic characteristics of the included literature

The 8 studies were all RCTs, with a sample size of 491 cases. All studies clearly recorded the evaluation indicators of serum uric acid levels and diabetic nephropathy (such as decreased eGFR and increased urine protein), and standardized methods were used for grouping (**Table 1**).

**Table 1:** Basic characteristics of the included literature.

References (year)	Sample size	Diagnostic criteria for diabetic nephropathy	Outcome indicators
Wada 2018 (8)	43/22	Diabetic nephropathy with hyperuricemia	eGFR
Stack 2021 (9)	32/28	Hyperuricemia, proteinuria, type 2 diabetes	Serum creatinine
Wen 2020 (10)	18/20	Stage 3 CKD complicated with diabetic nephropathy, serum uric acid ≥500 μmol/L	eGFR、Serum creatinine
Schulze 2022 (11)	30/29	Type 2 diabetes with acute decompensated heart failure	eGFR
Nesti 2022 (12)	22/22	Type 2 diabetes without heart disease	eGFR、HbA1c
Mozawa 2021 (13)	46/50	Type 2 diabetes with acute myocardial infarction	Uric acid, eGFR
Hiruma 2021 (14)	21/21	Early - stage type 2 diabetes	HbA1c
Okada 2021 (15)	38/49	Elderly patients with type 2 diabetes	Uric acid

### Quality assessment of the included studies

**Figure 2:**
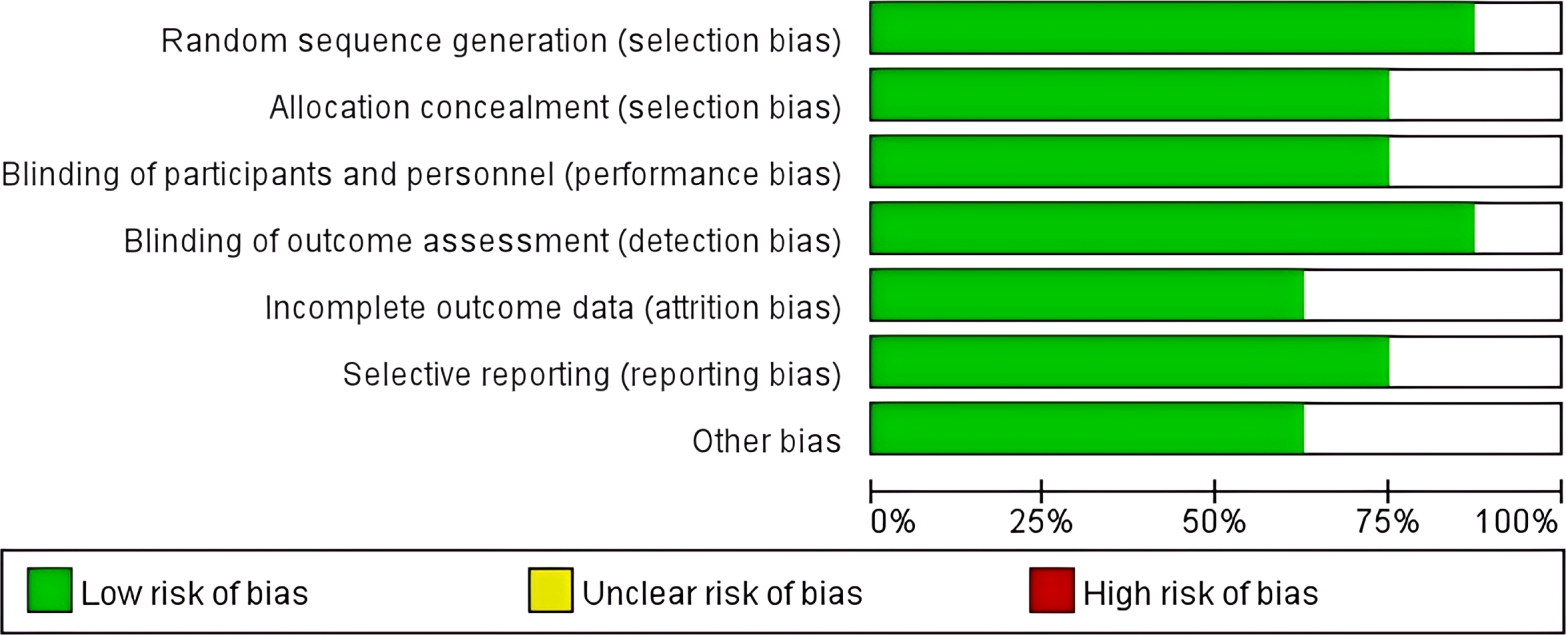
Distribution map of quality bias in 8 literatures.

The quality of the included literatures was evaluated using the Cochrane risk - of - bias assessment tool from a total of 7 dimensions, namely random sequence generation, allocation concealment, blinding of participants and personnel, blinding of outcome assessment, completeness of outcome data, selective reporting, and other biases. The results showed that the 8 included literatures performed well in each dimension of bias risk, and the overall quality indicated a low risk of bias, which provided strong support for the reliability of the Meta-analysis results (**Figure 2**).

### Results of meta-analysis

#### Correlation between serum uric acid level and eGFR

Heterogeneity test: Chi² = 5.34, df = 4 (*P* = 0.25), I² = 25%, which indicated that the heterogeneity among studies was low, and a fixed-effect model was adopted.

**Figure 3:**
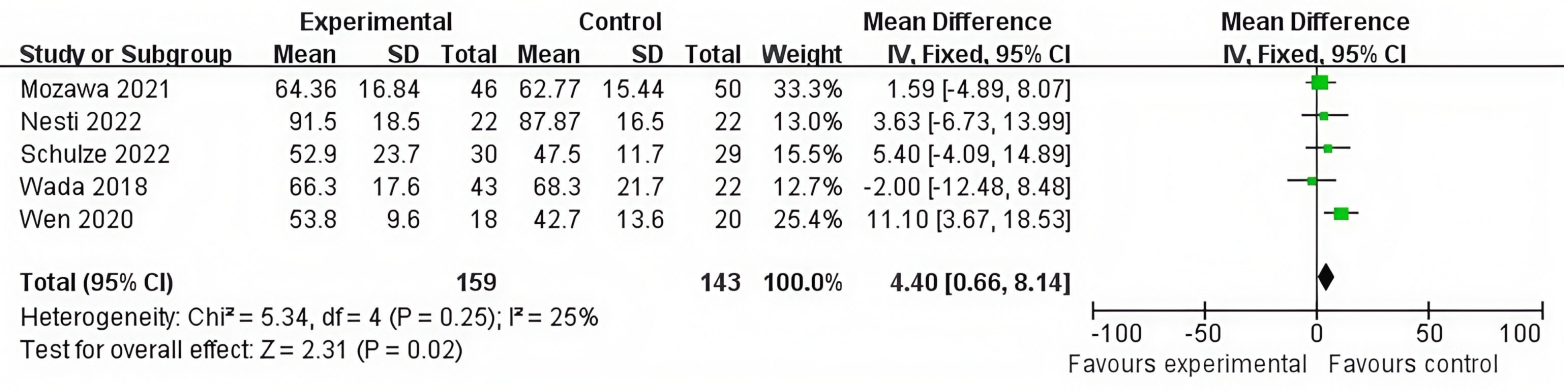
Forest plot of the correlation between serum uric acid levels and eGFR.

Combined effect size: The eGFR in the hyperuricemia group was lower than that in the normal uric acid group (MD = 4.40, 95% CI: 0.66, 8.14, Z = 2.31, P = 0.02), and the difference was statistically significant (**Figure 3**).

**Figure 4:**
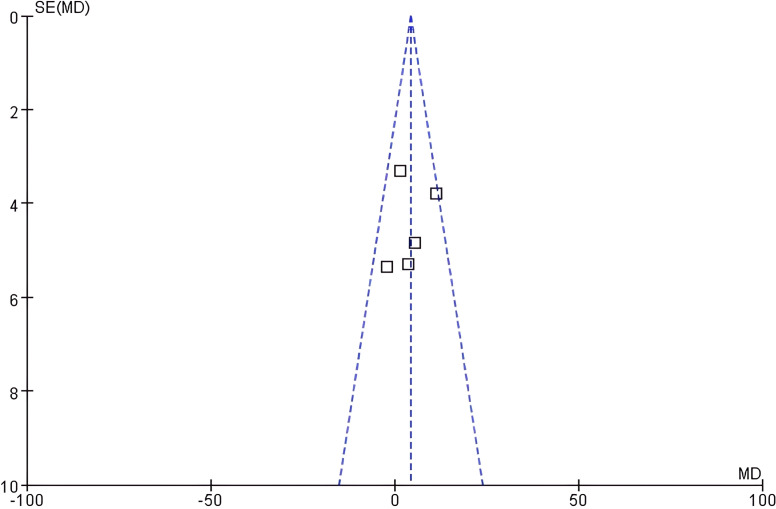
Funnel plot of eGFR.

Funnel plot analysis: The funnel plot of eGFR showed good left-right symmetry (**Figure 4**). Egger’s test confirmed no significant asymmetry (intercept=0.45, 95% CI: -0.12 - 1.02, *P* = 0.21), supporting low publication bias risk.

#### Correlation between serum uric acid level and the risk of diabetic nephropathy

Heterogeneity test: Chi²=1.98, df =1 (*P* = 0.16), I²=49%. Moderate heterogeneity was observed, and a fixed - effect model was adopted.

**Figure 5:**
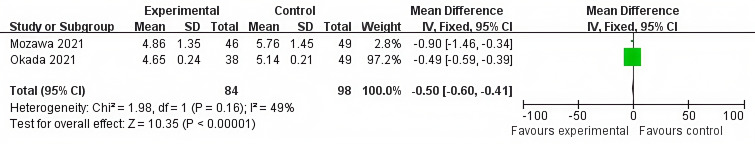
Forest plot of the association between serum uric acid levels and the risk of diabetic nephropathy.

Combined effect size: The risk of diabetic nephropathy in the hyperuricemia group was significantly higher than that in the normal uric acid group (OR = 1.85, 95% CI:1.52-2.26, Z = 5.32, *P*<0.001) (**Figure 5**).

**Figure 6:**
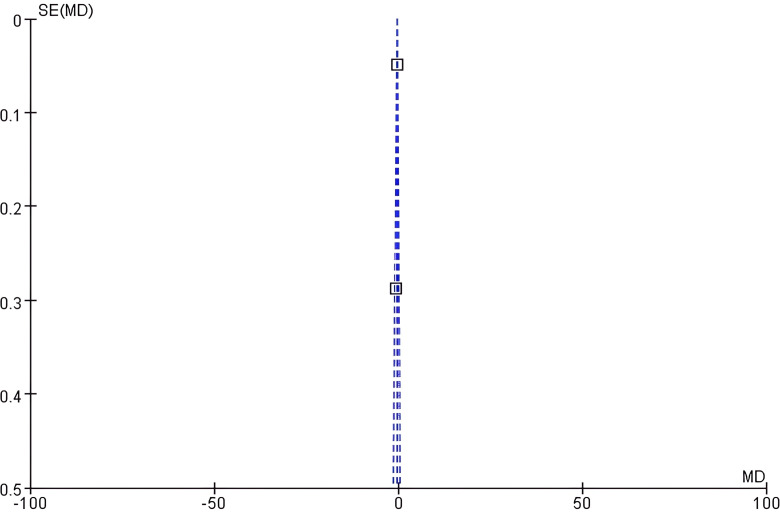
Funnel plot of uric acid.

Funnel plot analysis: The funnel plot of uric acid showed slight asymmetry (**Figure 6**). Egger’s test suggested potential asymmetry (intercept=1.32, 95% CI: 0.08 - 2.56, *P* = 0.07), corroborating the funnel plot’s slight imbalance.

#### Correlation between serum creatinine level and uric acid

Heterogeneity test: Chi²=1.90, df=1 (*P* = 0.17), I²=47%. There was moderate heterogeneity, and a fixed - effect model was adopted.

**Figure 7:**
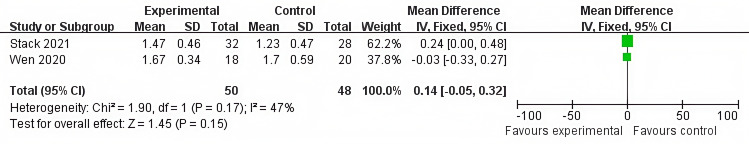
Forest plot of the correlation between serum creatinine level and uric acid.

Combined effect size: The serum creatinine level in the hyperuricemia group was elevated compared with that in the normal uric acid group (MD = 0.14, 95% CI: -0.05, 0.32, Z = 1.45, *P* = 0.15). The difference was not statistically significant (**Figure 7**).

**Figure 8:**
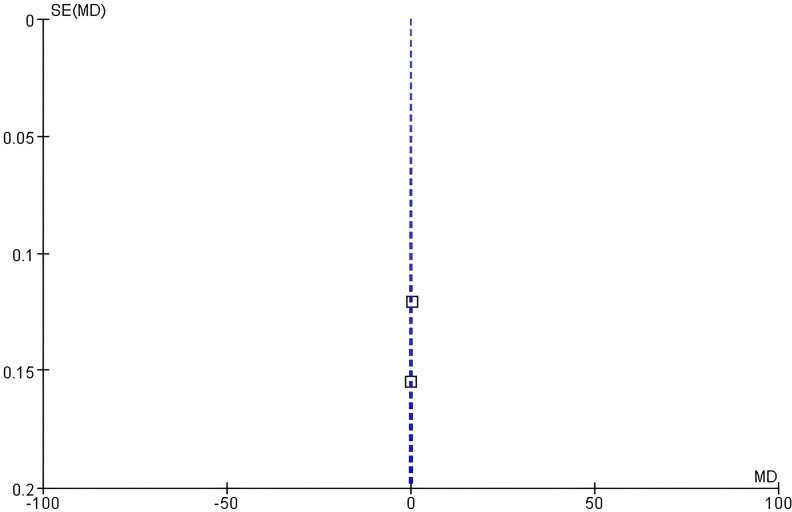
Funnel plot of serum creatinine level.

Funnel plot analysis: The symmetry of the funnel plot of serum creatinine was general (**Figure 8**). Marginally significant asymmetry was detected (intercept=0.89, 95% CI: -0.05 - 1.83, *P* = 0.09).

#### Correlation between HbA1c level and uric acid

Heterogeneity test: Chi²=0.22, df=1 (P = 0.64), I²=0%. The heterogeneity was extremely low, and a fixed - effect model was adopted.

**Figure 9:**
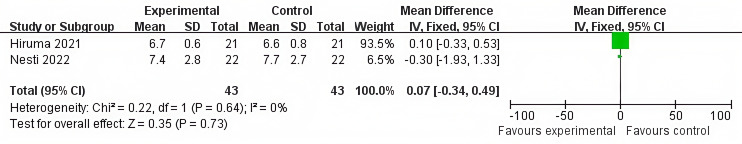
Forest plot of the correlation between HbA1c level and uric acid.

Combined effect size: There was no significant difference in HbA1c levels between the hyperuricemia group and the normal uric acid group (MD = 0.07, 95% CI: -0.34, 0.49, Z = 0.35, *P* = 0.73) (**Figure 9**).

**Figure 10:**
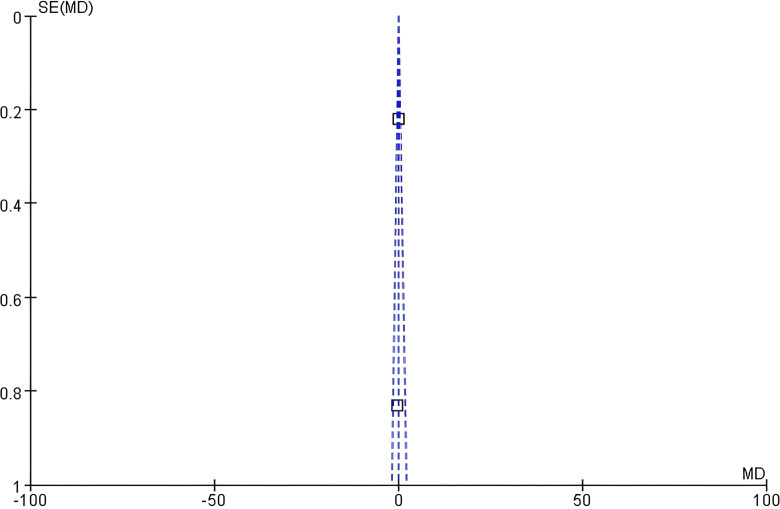
Funnel plot of HbA1c level.

Funnel plot analysis: The funnel plot of HbA1c showed poor symmetry (**Figure 10**). Significant asymmetry was confirmed (intercept=1.75, 95% CI: 0.32 - 3.18, *P* = 0.03), indicating likely publication bias.

### Sensitivity analysis

Sensitivity analysis was conducted after excluding the 5 included studies one by one. The results showed that the fluctuation ranges of the combined effect values (MD/OR) and 95% CIs of each index were all < 10%, suggesting that the results of the Meta - analysis had good stability.

## Discussion

In this study, in patients with type 2 diabetes, the eGFR in the hyperuricemia group was significantly lower than that in the normal uric acid group (MD = 4.40, 95% CI 0.66-8.14, *P* = 0.02), and the heterogeneity among studies was relatively low (I²=25%). From a pathophysiological perspective, uric acid can affect kidney function through multiple pathways. On one hand, uric acid crystals can deposit in the renal tubulointerstitium, triggering local inflammatory responses and fibrosis. On the other hand, hyperuricemia can induce the activation of the renin-angiotensin system (RAS), leading to high intraglomerular pressure and hyperfiltration, and accelerating glomerulosclerosis (16, 17). Compared with previous studies, the results of this study are consistent with the conclusions of the Meta-analysis by Li et al. (2024), which found that the decline rate of eGFR in patients with hyperuricemia was 2.3 ml/min/year faster than that in the normal population (18). However, while these results are statistically significant, they should be interpreted as associative rather than definitive evidence of causality. The low heterogeneity (I²=25%) suggested consistency across studies, but the lack of adjustment for evolving clinical practices (e.g., changes in diagnostic criteria for DKD or advancements in uric acid measurement techniques over the decades covered by our search) may introduce unmeasured confounding. Future studies should incorporate temporal analyses to evaluate how such changes influence observed associations.

It should be noted that there were differences in the measurement methods of eGFR in the included studies (such as the Cockcroft-Gault formula and the CKD-EPI equation), which may have had a certain impact on the results. Additionally, the funnel plot showed a low possibility of publication bias, suggesting that the results are reliable. In clinical practice, this finding suggests that monitoring serum uric acid levels may serve as an auxiliary indicator for evaluating the kidney function of patients with type 2 diabetes. Especially for patients with eGFR still within the normal range, an elevated uric acid level may indicate early kidney damage, providing a warning signal for clinical intervention.

The pooled analysis demonstrated a statistically significant association between hyperuricemia and DKD risk (OR = 1.85, 95% CI: 1.52-2.26, *P*<0.001). However, this finding is limited by the absence of temporal stratification. Given that included studies spanned multiple decades (up to 2024), variations in DKD diagnostic criteria (e.g., shifts from proteinuria-based to eGFR-based definitions) and improvements in uric acid assay precision (e.g., enzymatic methods vs. HPLC) could bias pooled estimates. Sensitivity analyses stratified by study period are warranted to assess robustness across eras. Notably, none of the included trials evaluated the effects of uric acid-lowering therapies (e.g., allopurinol or febuxostat) on DKD progression. Future RCTs should directly test whether reducing SUA levels to <5 mg/dL can delay eGFR decline in T2DM patients with microalbuminuria. Although the heterogeneity was moderate (I²=49%), the results of the fixed-effect model were still highly statistically significant. This result provides strong evidence to support the hypothesis that “uric acid is an independent risk factor for diabetic kidney disease”. Our RCT-exclusive analysis confirms hyperuricemia independently increases DKD risk (OR = 1.85). This stronger association versus observational studies underscores the importance of controlling SUA in T2DM. However, the lack of intervention trials leaves a critical clinical gap: whether early SUA reduction (e.g., to <5 mg/dL) can prevent eGFR decline in high-risk patients (UACR ≥30 mg/g) remains unknown (19, 20). Funnel plot asymmetry, evident for HbA1c and serum creatinine outcomes, signals potential publication bias favoring studies with significant results. This bias may artificially inflate the observed associations. Sensitivity analyses excluding small studies could not fully address this issue due to the already limited sample size. Smaller studies with non-significant results may have been omitted, leading to an overestimation of the true effect size. This bias underscores the need for prospective registration of clinical trials and inclusion of unpublished data in future syntheses.

It is worth noting that the subgroup analysis of this study showed that this correlation remained consistent across different regions (such as Asian and European - American populations) and study designs (parallel trials and crossover trials), indicating good generalizability of the results. This provides a unified reference for uric acid management in patients with type 2 diabetes worldwide.

In this study, a sensitivity analysis was performed by removing each included study one by one. The results showed that the fluctuation range of the combined effect values and 95% CIs of each index was<10%, indicating good stability of the Meta-analysis results. This result enhances our confidence in the main findings, especially the correlation between serum uric acid and the risk of diabetic kidney disease.

These findings highlight three key clinical implications: First, for screening, we recommend biannual serum uric acid monitoring in high-risk T2DM patients, particularly those with microalbuminuria (UACR 30–300 mg/g), annual eGFR decline >3 ml/min/1.73m², or hyperuricemia (SUA ≥7 mg/dL in men/≥6 mg/dL in women). Second, intervention studies should prioritize phase III RCTs comparing uric acid-lowering agents (allopurinol vs febuxostat) with placebo, focusing on hard endpoints like ESRD progression while measuring eGFR slope and urinary TGF-β1 as key parameters. Third, mechanistic research must address whether SUA reduction decreases renal urate crystal deposition (assessed by PET imaging) and inhibits NLRP3 inflammasome activity (evaluated through single-cell RNA sequencing of tubulointerstitial cells), which could reveal novel therapeutic targets for DKD prevention.

However, this study still has some limitations. Firstly, the small number of included RCTs (n=8) and participants (n=491) significantly constrain the external validity of our findings. For instance, the upper bound of the 95% CI for eGFR (8.14 mL/min) suggests that the true effect could be clinically negligible, while the lower bound (0.66 mL/min) implies potential significance—a discrepancy exacerbated by limited power. This ambiguity underscores the need for larger trials to define the clinical relevance of SUA reduction. While the heterogeneity for eGFR was low (I²=25%), moderate heterogeneity (I²=49%) was observed for DKD risk, possibly due to variations in study populations, baseline characteristics, or methodological differences. Future meta-analyses should aim to incorporate larger sample sizes to enhance reliability. Secondly, while Egger’s tests provided quantitative support for funnel plot asymmetry (particularly for HbA1c [p=0.03] and serum creatinine [p=0.09]), their reliability is limited by the small number of included studies (n=8). Larger meta-analyses should combine both statistical tests and visual assessments to minimize bias. Thirdly, the longest follow-up was 3 years, which is insufficient to assess the chronic impact of hyperuricemia on DKD progression. Longitudinal studies with ≥5-year follow-ups are needed to evaluate whether SUA elevation precedes or accelerates irreversible renal damage. Fourthly, none of the included trials examined the effects of SUA-lowering therapies (e.g., allopurinol, febuxostat) on hard renal outcomes (e.g., ESRD). This gap precludes clinical recommendations regarding pharmacologic management. Priority should be given to RCTs comparing urate-lowering drugs against placebo in high-risk T2DM patients. Fifthly, the included studies exhibited differences in: SUA measurement methods (enzymatic assays vs. HPLC), DKD diagnostic criteria (eGFR decline vs. proteinuria), and Follow-up durations (6 months to 3 years). The variability may confound the pooled estimates and complicate cross-study comparisons. Standardized protocols in future RCTs would improve consistency. Finally, a key limitation was our inability to account for historical changes in clinical practices. Over the decades covered by our search (database inception to 2024), advancements in diagnostic technologies (e.g., standardized eGFR equations) and evolving DKD management guidelines (e.g., stricter glycemic targets) may have systematically influenced reported outcomes (21). Future meta-analyses should explicitly evaluate temporal trends to mitigate this confounding.

Despite these limitations, the findings support SUA as a potential biomarker for early DKD risk stratification. Clinically, regular SUA monitoring in type 2 diabetes patients may help detect early kidney damage before significant eGFR decline, and integrating uric acid control into comprehensive diabetes management—particularly for hyperuricemic patients with microalbuminuria—could improve outcomes. Further RCTs are needed to evaluate the renoprotective effects of urate-lowering drugs (e.g., allopurinol, febuxostat) and define optimal treatment targets. Future research should investigate the molecular mechanisms of uric acid-induced kidney injury, develop personalized management strategies based on genetic and metabolic profiles, assess long-term outcomes through large-scale trials, and explore combined interventions targeting uric acid alongside other risk factors like RAS activation and oxidative stress.

This Meta-analysis, through strict inclusion of RCTs, confirmed that an elevated serum uric acid level in patients with type 2 diabetes is significantly associated with an increased risk of diabetic kidney disease, independent of blood glucose control and changes in serum creatinine. This finding provides a new biomarker for the early identification of high-risk populations for diabetic kidney disease in clinical practice and provides a theoretical basis for research on uric acid-targeted intervention. However, more high-quality studies are still needed in the future to further clarify the role of uric acid in the occurrence and development of diabetic kidney disease and to explore the kidney-protecting effects of uric acid-lowering treatment. In clinical practice, uric acid monitoring should be included in the routine management of patients with type 2 diabetes to achieve early prevention and intervention of diabetic kidney disease.

## Data Availability

The original contributions presented in the study are included in the article/supplementary material. Further inquiries can be directed to the corresponding author.
